# Immunophenotyping of Circulating and Intratumoral Myeloid and T Cells in Glioblastoma Patients

**DOI:** 10.3390/cancers14235751

**Published:** 2022-11-23

**Authors:** Sascha Marx, Fabian Wilken, Lea Miebach, Mikael Ispirjan, Frederik Kinnen, Sebastian Paul, Sandra Bien-Möller, Eric Freund, Jörg Baldauf, Steffen Fleck, Nikolai Siebert, Holger Lode, Andreas Stahl, Bernhard H. Rauch, Stephan Singer, Christoph Ritter, Henry W. S. Schroeder, Sander Bekeschus

**Affiliations:** 1 Department of Cancer Immunology and Virology, Dana-Farber Cancer Institute, Harvard Medical School, 450 Brookline Avenue, Boston, MA 02215, USA; 2 Department of Neurosurgery, Greifswald University Medical Center, Ferdinand-Sauerbruch-Str., 17475 Greifswald, Germany; 3 ZIK *plasmatis*, Leibniz Institute for Plasma Science and Technology (INP), Felix-Hausdorff-Str. 2, 17489 Greifswald, Germany; 4 Department for General, Thoracic, Vascular, and Thorax Surgery, Greifswald University Medical Center, Ferdinand-Sauerbruch-Str., 17475 Greifswald, Germany; 5 Department of Pharmacology, C_DAT, Greifswald University Medical Center, Felix-Hausdorff-Str. 3, 17489 Greifswald, Germany; 6 Department of Ophthalmology, Greifswald University Medical Center, Ferdinand-Sauerbruch-Str., 17475 Greifswald, Germany; 7 Department of Pediatric Oncology, Greifswald University Medical Center, Ferdinand-Sauerbruch-Str., 17475 Greifswald, Germany; 8 Pharmacology and Toxicology, Department of Human Medicine, University of Oldenburg, Carl-von-Ossietzky-Str. 9-11, 26129 Oldenburg, Germany; 9 Department of Pathology, Greifswald University Medical Center, Ferdinand-Sauerbruch-Str., 17475 Greifswald, Germany; 10 Department of Pathology and Neuropathology, Tuebingen University Medical Center, Liebermeisterstr. 8, 72076 Tuebingen, Germany; 11 Institute of Clinical Pharmacy, Greifswald University, Felix-Hausdorff-Str. 3, 17489 Greifswald, Germany

**Keywords:** CD163, GBM, glioma, macrophages, PD1, PSGL-1, T cells

## Abstract

**Simple Summary:**

Immune-therapeutical approaches still are not as impactful in glioblastoma (GBM) as in other types of cancer. Due to its unique pathoanatomical localization behind the bony skull, GBM samples are not as easy to obtain, so understanding the immuno-phenotypes in GBM is challenging. Here we present a thorough characterization of the immune status in the GBM tumor microenvironment (TME) and the circulation of the patients compared to a matched proband cohort.

**Abstract:**

Glioblastoma is the most common and lethal primary brain malignancy that almost inevitably recurs as therapy-refractory cancer. While the success of immune checkpoint blockade (ICB) revealed the immense potential of immune-targeted therapies in several types of cancers outside the central nervous system, it failed to show objective responses in glioblastoma patients as of now. The ability of glioblastoma cells to drive multiple modes of T cell dysfunction while exhibiting low-quality neoepitopes, low-mutational load, and poor antigen priming limits anti-tumor immunity and efficacy of antigen-unspecific immunotherapies such as ICB. An in-depth understanding of the GBM immune landscape is essential to delineate and reprogram such immunosuppressive circuits during disease progression. In this view, the present study aimed to characterize the peripheral and intratumoral immune compartments of 35 glioblastoma patients compared to age- and sex-matched healthy control probands, particularly focusing on exhaustion signatures on myeloid and T cell subsets. Compared to healthy control participants, different immune signatures were already found in the peripheral circulation, partially related to the steroid medication the patients received. Intratumoral CD4+ and CD8+ TEM cells (CD62L^low^/CD45RO^high^) revealed a high expression of PD1, which was also increased on intratumoral, pro-tumorigenic macrophages/microglia. Histopathological analysis further identified high PSGL-1 expression levels of the latter, which has recently been linked to increased metastasis in melanoma and colon cancer via P-selectin-mediated platelet activation. Overall, the present study comprises immunophenotyping of a patient cohort to give implications for eligible immunotherapeutic targets in neurooncology in the future.

## 1. Introduction

Immune-therapeutical approaches revolutionized the treatment and prognosis of several types of cancer outside the central nervous system (CNS) in the past decade [1,2,3,4]. Given the poor prognosis and limited treatment options for brain malignancies and glioblastoma, immunotherapy poses a promising therapeutic avenue in neurooncology. However, blockade of the immune checkpoint programmed death (PD1) widely failed to show objective responses in glioblastoma patients in a clinical trial recently (NCT 02017717) [5]. Only a small subset of patients (8%) with temozolomide-induced hypermutations benefited from ICB, highlighting the need for in-depth knowledge of the distinct immune landscape in glioblastoma patients to broaden the range of patients benefiting from such an approach.

Shielded from the peripheral circulation by the blood-brain barrier (BBB) to avoid collateral damage following immune cell entry and attack, the brain comprises a unique immune compartment and is considered one of the “immune privileged” organs of our body [6]. This concept has been partially revised since the presence of functional lymphatic vasculature has been reported along the dural sinuses in mice [7,8] and the notion that CNS-derived antigens can elicit immune responses in cervical lymph nodes [9]. While T cells are not abundant in the brain, myeloid lineage cells comprise 30% of tissue-resident cells [10]. They represent a complex, heterogeneous, and dynamic population of yolk sac-derived microglia in the parenchyma, and border-associated macrophages, partially originating from the bone marrow. In neuropathological disorders, the BBB is often compromised, resulting in increased infiltration of multiple immune cell types from the peripheral circulation at later stages [11]. The immunosuppressive microenvironment in glioblastoma is associated with a high prevalence of pro-tumorigenic glioma-associated macrophages/microglia in the tumor, facilitating GBM invasion, growth, and angiogenesis [12]. In addition, glioblastoma cells propagate multiple modes of T cell dysfunction, including T cell anergy [13], tolerance [14], sequestration [15], senescence, and exhaustion [16]. Compared to melanoma or non-small cell lung cancers, glioblastoma cells exhibit a lower burden of somatic mutations, low-quality neoantigens, and poor antigen priming, which further impairs anti-tumor immunity potency [6].

The present study is the first to comprise a flow cytometric immunophenotyping of the peripheral and intratumoral immune compartment of 28 glioblastoma patients compared to age- and sex-matched healthy control participants with a particular focus on exhaustion signatures on circulating monocytes, glioma-associated macrophages/microglia, and circulating and intratumoral T cells. The myeloid cell landscape was further characterized by histopathological analysis in 19 patients and compared to healthy control tissue. Our findings provide a deeper understanding of immune signatures in glioblastoma patients to help identify potential targets for immunotherapies in the future.

## 2. Materials and Methods

### 2.1. Patient Cohorts

**Figure 1:**
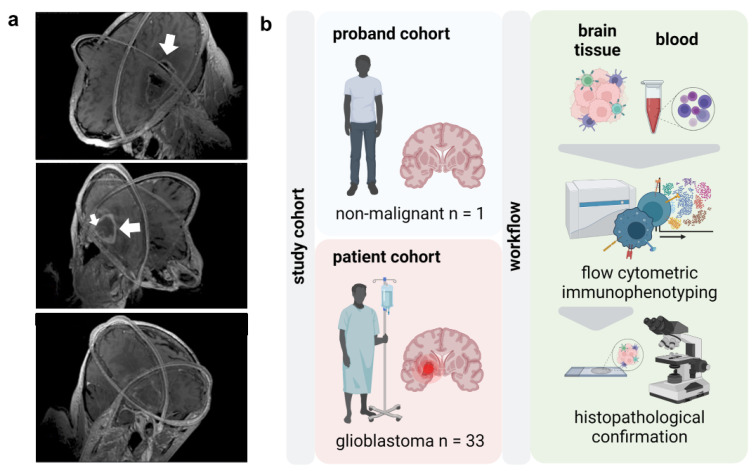
**Study overview.** (**a**) 3D visualizations of MRI scans of glioblastoma (white arrows) patients reconstructed from axial, sagittal, and coronal slices; (**b**) study overview.

The local ethics committee approved the study (BB089/08b) and written informed consent was obtained from every participating GBM patient and control participant. The cohort comprises 35 patients (23 male, 12 female, mean age 68, ranging from 41 to 87 years) (Figure 1a and Table A1). To characterize the peripheral myeloid and lymphoid immune compartment, EDTA anticoagulated blood was withdrawn from 28 glioblastoma patients on the day before surgery. Immunoprofiling of intratumoral immune cells was done in tumors of 12 patients after neurosurgical resection. Histopathological confirmation was available for 19 patients for IHC analysis of FFPE-embedded tumor samples (Table 1 and Figure A2). Age- and sex-matched healthy control subjects were recruited from the local ophthalmology department’s elective surgical candidates (Figure 1b).

### 2.2. Sample Processing

PBMCs were isolated by density gradient centrifugation and staining for flow cytometry was done immediately after. Surgically resected glioblastoma tissues were mechanically disrupted into small pieces with a disposable, sterile scalpel and further dissociated into single-cell suspension using the enzymatic brain tumor dissociation kit (P) from Milteny Biotec following the manufacturer’s protocol. Tumor samples were immediately stained for consecutive flow cytometric analysis.

### 2.3. Flow Cytometry

**Figure A1:**
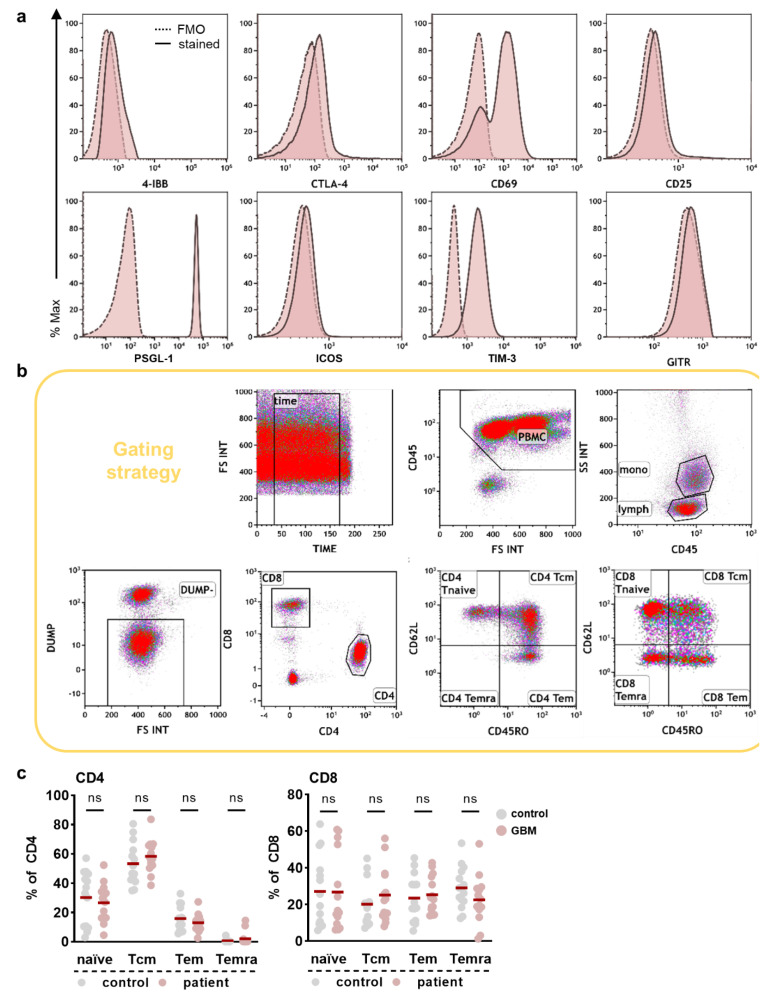
**Immunophenotyping of PBMCs in glioblastoma patients and healthy individuals.** (**a**) Flow cytometry histograms showing isotype controls and stained circulating myeloid cells; (**b**) representative gating strategy for identification of myeloid and T cell subsets; (**c**) Circulating T cell subset frequencies in glioblastoma patients compared to healthy controls. Statistical analysis was performed using paired *t*-test. ns = non-significant. cm = central memory. em = effector memory. emra = effector memory expressing CD45RA.

PBMC and tumor cell suspensions from 16 glioblastoma patients were stained with the same core panel and antibodies targeting CD1c, CD3, CD4, CD8a, CD14, CD15, CD16, CD45, CD45-RO, CD56, and CD62L. Beyond that core panel to identify subpopulations, PBMC were stained with antibodies targeting CD25, CD69, 4-IBB (CD137), CTLA4 (CD152), ICOS (CD278), PD1 (CD279), GITR (CD357), TIM3 (CD366), and PSGL-1 (CD162) (all BioLegend, Amsterdam, The Netherlands). With regard to the prominent role of myeloid cells in glioblastoma, PBMCs of 11 patients were separately stained with antibodies targeting CD11b, CD14, CD45, CD55, CD97, PSGL-1 (CD162), CD163, CD169, CD204, CD273, CD276, HLA-ABC, and HLA-DR as well as DAPI for identifying dead cells (all Miltenyi Biotec, Teterow, Germany). Tumor samples of 12 patients were antibody-stained against the target antigens CD3, CD4, CD8, CD62L, CD45, CD45RO, PSGL-1 (CD162), PD1 (CD279), and TIM3 (CD366). Zombie-NIR was used for live-dead discrimination (all BioLegend) (Table A2). FMOs were prepared to correct for the antibodies’ fluorochromes fluorescence spillover. Samples were analyzed on a 3-laser, 10-color Gallios flow cytometer (Beckman-Coulter, Krefeld, Germany). Data analysis was performed using Kaluza software 2.1.3 (Beckman-Coulter) based on the gating strategy shown (Figure A1).

### 2.4. Immunohistochemistry

For immunohistochemistry-staining, 10 µM paraffin sections were deparaffinized in Xylol, rehydrated, and boiled for 5 min in citrate buffer (pH = 6) for antigen retrieval. Before imaging by light microscopy, sections were incubated with antibodies targeting CD68, PSGL-1 (CD162), and CD163 (dilution 1:50; Enzo Life Sciences, Lörrach, Germany) overnight at 4 °C. After washing, sections were incubated with an immuno-peroxidase polymer for 20 min and mounted on glass microscopy slides using mounting medium (ibidi, Gräfelfing, Germany). Imaging and analysis were performed with a high-content imaging device (Operetta CLS) and its associated software (Harmony 4.9; both PerkinElmer, Hamburg, Germany). For the measurement of non-fluorescent immunohistochemical staining, 3 channels with the following settings were used: brightfield (exposure time: 20 ms, power 50%), a pseudo-absorption channel (exposure time: 5 ms, power 5%), and a tissue autofluorescence channel (exposure time: 100 ms, power 50%). The scanned image was inverted in the first and second channels and merged into one channel. As the size of the entire section was calculated, the number of positive cells per mm² could be determined using quantitative image-based object segmentation by unsupervised computer algorithms.

### 2.5. Statistical Analysis

Statistical analysis was performed with Prism 9.4.1 (GraphPad Software, San Diego, CA, USA), and details are given in the figure legends. Levels of significance are indicated as follows: *p* = 0.05 (*), *p* = 0.01 (**), *p* = 0.001 (***), ns = non-significant. 

## 3. Results

### 3.1. Alterations in the Peripheral Myeloid and Lymphoid Compartment in Glioblastoma Patients

Glioblastoma shows a profoundly dysfunctional anti-tumor immunity and remains refractory to immunotherapy. Delineating the glioblastoma immune landscape is essential to target immunosuppressive circuits in this disease. In this light, the present study aimed to perform thorough immunophenotyping of the peripheral and intratumoral immune compartments in glioblastoma tissues of patients (Figure 1a) compared to age- and sex-matched healthy individuals. Flow cytometric analysis was performed of peripheral blood mononuclear cells (PBMCs) and tumor-infiltrating leucocytes (TILs) following histopathological glioblastoma confirmation (Figure 1b). 

**Figure 2:**
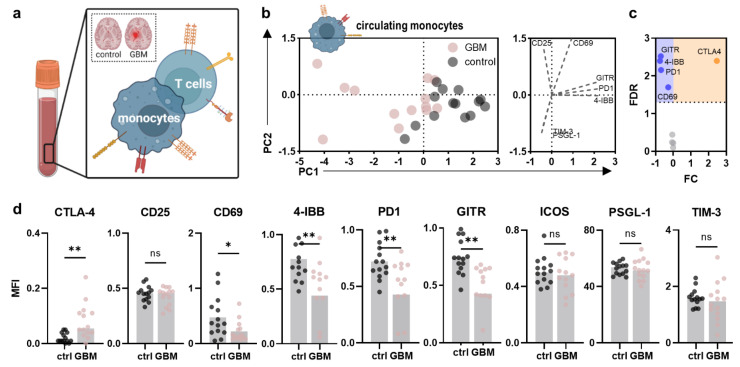
**Alterations in the peripheral myeloid immune compartment of glioblastoma patients compared to healthy controls.** (**a**) schematic overview of immunophenotyping analysis of circulating monocytes and T cells in the present study; (**b**) principal component analysis (PCA) calculated from marker expression profiles of circulating monocytes in age- and sex-matched healthy control participant (control) and glioblastoma patient (GBM)-derived PBMC showing PC scores (**left**) and loadings (**right**); (**c**) volcano plot showing significantly (dotted line, *p* < 0.05) up- (orange) and downregulated (blue) markers on circulating monocytes of glioblastoma patients compared to healthy individuals; (**d**) mean fluorescence intensities (MFI) of CTLA4, CD25, CD69, 4-IBB, PD1, GITR, ICOS, PSGL-1, and TIM-3 analyzed on circulating monocytes in glioblastoma patients and healthy individuals’ PBMC. Bar graphs show mean ± standard deviation of the mean (SEM). Statistical analysis was performed using paired *t*-test (* *p* < 0.05, ** *p* < 0.01). ns = non-significant. ctrl = healthy control participants. GBM = glioblastoma patients. PC = principal component. FC = fold change. FDR = false discovery rate.

**Figure A2:**
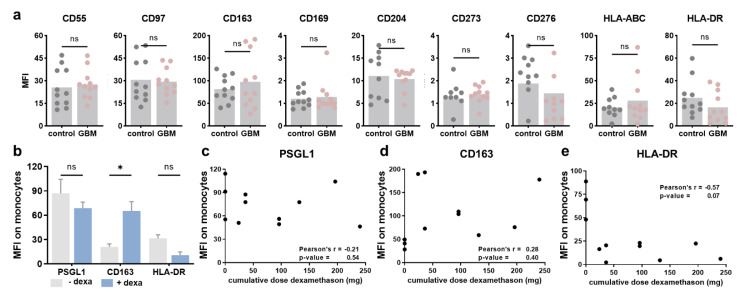
**Immunophenotyping of circulating monocytes and impact of steroid medication.** (**a**) mean fluorescence intensities (MFI) of CD55, CD97, CD163, CD169, CD204, CD273, CD276, HLA-ABC, and HLA-DR on circulating monocytes of glioblastoma patients (GBM) compared to healthy individuals; (**b**) quantification of PSGL-1, CD163, and HLA-DR expression on circulating monocytes in GBM patients with Dexamethasone medication or without; (**c**–**e**) Pearson’s correlation of PSGL-1 (**c**), CD163 (**d**), or HLA-DR (**e**) expression with the cumulative doses of dexamethasone given to the GBM patients prior to blood withdrawal. Statistical analysis was performed using paired *t*-test (* *p* < 0.05). ns = non-significant. dexa = dexamethasone.

PBMC activation and immunosuppressive signatures were evaluated, focusing on circulating monocytes and T cells (Figure 2a). Principal component analysis (PCA) of surface marker expression profiles of circulating monocytes already outlined a distinct pattern in glioblastoma patients compared to control participants, as indicated by a markedly different principal component (PC) 1. Hereof, differences were mainly linked to increased expression of the immune checkpoint molecules glucocorticoid-induced TNFR-related protein (GITR; CD357), programmed death 1 (PD1; CD279), the activation-induced costimulatory molecule 4-IBB (CD137), and CD69 in healthy control participants as indicated by their correlation with PC1 displayed in the loading plot (Figure 2b). Among all markers investigated, the immune checkpoint cytotoxic T lymphocyte-associated protein 4 (CTLA4; CD152) was the only marker found to be increased on circulating monocytes in glioblastoma patients (Figure 2c). No alterations in expression were found for CD25, inducible T cell costimulator (ICOS; CD278), selectin P-ligand (PSGL-1; CD162), T cell immunoglobulin and mucin-domain containing-3 (TIM-3; CD366) (Figure 2d), and others (Figure A2a). Despite adjuvant radiochemotherapy, symptomatic steroid treatment is part of the standard of care in glioblastoma patients to reduce commonly occurring brain edema. Steroids are known to significantly influence immune cell function and phenotype and alter the expression of PSGL-1, CD163, and HLA-DR on circulating monocytes of glioblastoma patients in the present study (Figure A2b). Yet, a correlation between expression patterns and the patient-received cumulative dexamethasone dose was not observed in any marker (Figure A2c–e).

**Figure 3:**
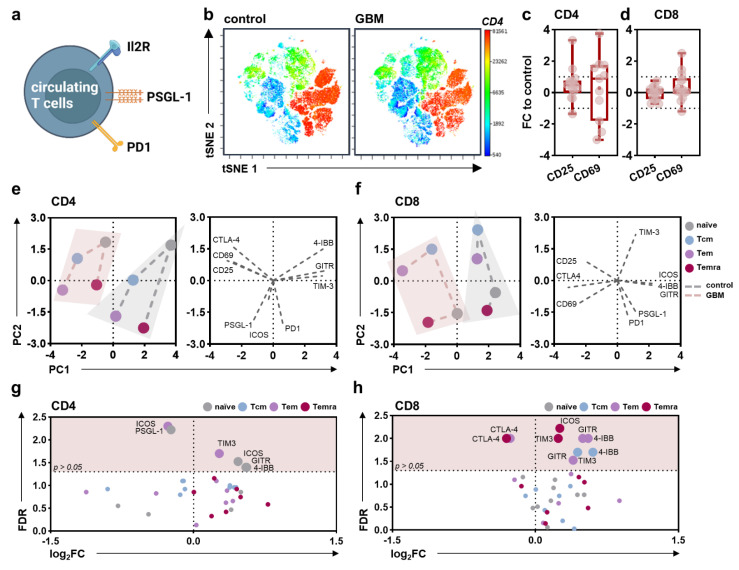
**Alterations in the peripheral lymphoid immune compartment of glioblastoma patients compared to healthy controls.** (**a**) schematic overview of immune marker expression profiling on circulating T cells; (**b**) t-distributed stochastic neighbor embedding (tSNE) calculated from flow cytometric analysis of marker expression on PBMCs isolated from healthy donors (control) and glioblastoma patients (GBM) showing z-scaled CD4 expression; (**c**,**d**) 5–95 percentile boxplots showing CD25 and CD69 expression on circulating CD4^+^ T helper cells (**c**) and CD8^+^ cytotoxic T cells (**d**) normalized to age- and sex-matched healthy control participants; (**e**,**f**) principal component analysis (PCA) calculated from marker expression on CD4^+^ (**e**) or CD8^+^ (**f**) naïve, central memory (cm), effector memory (em), and effector memory expressing CD45RA (emra) T cell subpopulations showing PC scores (**left**) and loadings (**right**); (**g**,**h**) volcano plots displaying markers differentially expressed on CD4^+^ (**g**) and CD8^+^ T_naïve_, T_cm_, T_em_, and T_emra_ cells. Statistical analysis was performed using paired *t*-tests. control = healthy control participants. GBM = glioblastoma patients. FC = fold change. PC = principal component. FDR = false-discovery rate.

Next, surface marker expression patterns were evaluated on circulating T cells (Figure 3a) and their subpopulations (naïve, central memory (cm), effector memory (em), effector memory expressing CD45RA (emra)) based on CD62L and CD45RO expression (Figure A2b)). No significant differences in the distribution of different T cell subpopulations in glioblastoma patients compared to healthy individuals could be observed (Figure A2c), as underlined by t-stochastical neighbor embedding (tSNE) analysis (Figure 3b). Likewise, a global difference in the activation status of CD4^+^ T helper (Figure 3c) and CD8^+^ cytotoxic T cells was not observed (Figure 3d). However, a distinct marker expression pattern was found for CD4^+^ T helper (Figure 3e) and CD8^+^ cytotoxic T cell subsets (Figure 3f) between PBMC of the patient and proband cohorts as indicated by different PC scores and underlying variables in the calculated PCA. Concerning CD4^+^ T helper cells, a distinct expression pattern was mainly found in CD62L^high^CD45RO^low^ naïve T cells and CD62L^low^CD45RO^high^ T_em_ cells. The former showed increased ICOS, GITR, and 4-IBB, and decreased PSGL-1 expression levels, while in the latter, we found TIM-3 to be increased and ICOS to be decreased (Figure 3g). In CD8^+^ cytotoxic T cells, differences were found for naïve, CD62L^high^CD45RO^high^ T_cm_, and CD62L^low^CD45RO^low^ T_emra_ cells with a main decrease in CTLA4, and an increase for TIM3, ICOS, GITR, and 4-IBB (Figure 3h).

### 3.2. Glioblastoma Shows Distinct Myeloid Expression Signatures Compared to Healthy Individuals

**Figure 4:**
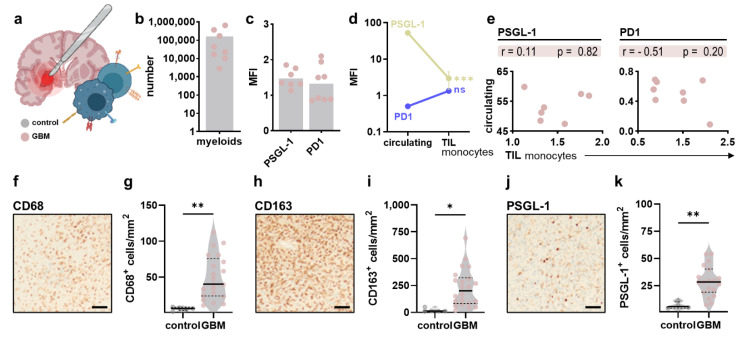
**Myeloid immune infiltration in glioblastoma tumors.** (**a**) schematic overview of intratumoral immune profiling after neurosurgical resection of tumor samples; (**b**) absolute numbers of myeloid cells in tumor samples; (**c**) surface expression of PSGL-1 and PD1 on intratumoral myeloid cells; (**d**) comparison of PSGL-1 and PD1 expression on circulating and tumor-infiltrating myeloid cells; (**e**) Pearson’s correlation of PSGL-1 and PD1 expression on circulating and tumor-infiltrating myeloid cells; (**f**–**k**) representative immunohistochemical images and quantification of CD68^+^ (**f**,**g**), CD163^+^ (**h**,**i**) and PSGL1^+^ cells (**j**,**k**) in control and malignant brain tissue. Bar graphs show mean + individual values. Statistical analysis was performed using paired *t*-test (* *p* < 0.05, ** *p* < 0.01, *** *p* < 0.001).

Shielded from the peripheral circulation to prevent unwanted immune cell entry and attack, the brain is considered one of our body’s “immune privileged” organs with a unique immune landscape mainly comprised of myeloid cells. After focusing on changes in the peripheral immune compartment of glioblastoma patients, the present study sought to identify alterations in immune signatures in the local GBM tumor immune infiltrate (Figure 4a) with a focus on the myeloid lineage (Figure 4b). Analysis of PSGL-1 and PD1 expression on intratumoral myeloid cells (Figure 4c) revealed a significant decrease in the latter compared to circulating monocytes (Figure 4d). However, when comparing individual patients, a significant correlation could not be identified (Figure 4e). Histopathological analysis was done to compare the myeloid infiltration in healthy and malignant brain tissue. Immunohistochemical staining of CD68 (Figure 4f) revealed a significant increase in CD68^+^ cells in the malignant parenchyma (Figure 4g). Similar results were obtained for CD163 (Figure 4h,i) and PSGL-1 (Figure 4j,k). 

### 3.3. Glioblastoma Shows High Infiltration of CD4^+^ and CD8^+^ CD62L^low^CD45RO^high^ Effector Memory T Cells

**Figure 5:**
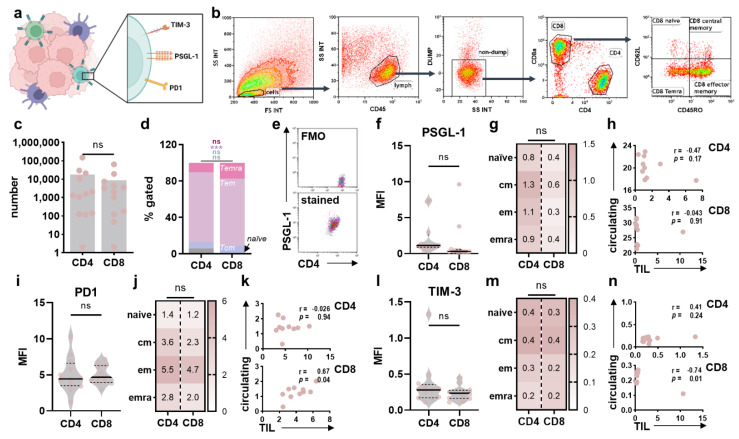
**Immunophenotyping of tumor-infiltrating T cells in glioblastoma tissues.** (**a**) schematic overview of marker expression profiling on intratumoral T cells in glioblastoma patients; (**b**) representative gating strategy to identify CD4^+^ and CD8^+^ T cells and their subpopulations in glioblastoma samples; (**c**) absolute numbers of CD4^+^ and CD8^+^ T cells; (**d**) frequency of CD4^+^ and CD8^+^ T_naïve_, T_cm_, T_em_, and T_emra_ cells; (**e**) representative flow cytometry dot plot graphing of FMO and anti-PSGL-1 stained CD4^+^ T cells; (**f**,**g**) surface expression of PSGL-1 on intratumoral CD4^+^ and CD8^+^ T cells (**f**) and T_naïve_, T_cm_, T_em_ and T_emra_ subsets (**g**); (**h**) Pearson’s correlation between PSGL-1 expression on circulating and intratumoral CD4^+^ and CD8^+^ T cells; (**i**,**j**) surface expression of PD1 on intratumoral CD4^+^ and CD8^+^ T cells (**i**) and T_naïve_, T_cm_, T_em_, and T_emra_ subsets (**j**); (**k**) Pearson’s correlation between PD1 expression on circulating and intratumoral CD4^+^ and CD8^+^ T cells; (**l**,**m**) surface expression of TIM-3 on intratumoral CD4^+^ and CD8^+^ T cells (**l**) and T_naïve_, T_cm_, T_em_, and T_emra_ subsets (**m**); (**n**) Pearson’s correlation between TIM-1 expression on circulating and intratumoral CD4^+^ and CD8^+^ T cells. Bar graphs show mean + individual values. Statistical analysis was performed using paired *t*-test (*** *p* < 0.001). ns = non-significant. FMO = fluorescence minus one. MFI = mean fluorescence intensity. cm = central memory. em = effector memory. emra = effector memory expressing CD45RA. TIL = tumor-infiltrating leucocytes.

Albeit less abundant in the glioblastoma tumor microenvironment (TME) compared to glioma-associated macrophages/microglia, T cells are a vital part of anti-tumor immune responses. Tumor-infiltrating CD4^+^ and CD8^+^ T cells (Figure 5a) were thus characterized using flow cytometric analysis (Figure 5b). No differences were observed in absolute numbers of CD4^+^ T helper compared to CD8^+^ cytotoxic T cells in the tumors (Figure 5c). Hereof, CD62L^low^CD45RO^high^ T_em_ comprised the dominant T cell subpopulation both in CD4^+^ T cells and CD8^+^ T cells, with a higher presence observed for the former (Figure 5d). Flow cytometric analysis of checkpoint and exhaustion signatures on T cells (Figure 5e) revealed no differences in PSGL-1 expression between CD4^+^ and CD8^+^ T cells (Figure 5f) and their subpopulations (Figure 5g). Similar to the myeloid compartment, a significant correlation was not observed when comparing expression levels with circulating T lymphocytes (Figure 5h). By contrast, although significant differences in PD1 expression on CD4^+^ and CD8^+^ T cells (Figure 5i) and their subpopulations (Figure 5j) were not identified, a strong positive correlation between PD1 expression on circulating and tumor-infiltrating CD8^+^ cytotoxic T cells could be observed (Figure 5k). Of note, CD4^+^ and CD8^+^ T_em_ cells showed a particularly strong expression of PD1 compared to their naïve counterparts. Similarly, TIM-3 expression was evaluated on CD4^+^ and CD8^+^ T cells (Figure 5l) and their subpopulations (Figure 5m) but did not differ significantly. Interestingly, a negative correlation was found between TIM3 expression on circulating and tumor-infiltrating CD8^+^ T cells (Figure 5n).

## 4. Discussion

The success of immunotherapeutic approaches such as ICB relies on the body’s ability to recognize tumor cells as foreign and mount T cell responses against tumor antigens. High rates of somatic mutations in cancer cells cause the occurrence of tumor-specific and cancer-associated neoantigens [17,18], recognizable by autologous T cells of the host. Glioblastoma is considered immunologically “cold” due to low-quality neoantigens, low-mutational load, poor antigen priming, and an overall immunosuppressive tumor microenvironment, limiting the efficacy of ICB markedly. Nonetheless, although only a small subset of patients (8%) profited from anti-PD1 checkpoint immuno-blockade in a recent clinical trial (NCT 02017717), responders showed an increased median overall survival compared to the standard of care, highlighting the potential of immune-targeted therapies also in treatment strategies of glioblastoma [19]. Understanding the distinct glioblastoma immune landscape, a tumor type evolving in one of the three “immune privileged” organs in the human body, is essential to expand the number of patients that could profit from immuno-oncological approaches. In this light, the present study comprises immunophenotyping of the peripheral and intratumoral immune compartments of 35 glioblastoma patients compared to age- and sex-matched healthy control participants based on flow cytometry and histopathological confirmation. 

Examining the immune status of glioblastoma patients has a high prognostic and predictive relevance to increasing immunotherapeutic success. Immune modulations in the peripheral immune compartment are closely linked to therapy response or progression [20]. In line with a slight downregulation of HLA-DR on circulating monocytes of glioblastoma patients observed in the present study, it has previously been reported that patients with newly diagnosed glioblastoma show an increased number of circulating CD33^+^ HLA-DR^−^ myeloid-derived suppressor cells (MDSCs) comprised of immature, monocytic and neutrophilic subsets [21]. Blood-derived neutrophilic and eosinophilic MDSCs are further considered to suppress autologous non-specific T cell proliferation and IFNγ secretion leading to impaired anti-tumor immunity in glioblastoma patients [22]. Compared to healthy individuals, circulating monocytes were found to have decreased activation and expression of costimulatory 4-IBB and GITR and upregulation of the immune checkpoint CTLA4. In an experimental model of Sjörgen’s disease, GITRL/GITR signaling was identified to reduce the suppressive function of MDSCs on T cell proliferation and release of suppressive factors, including arginase and NO, while promoting differentiation into mature myeloid cells [23]. Likewise, 4-IBB is a potent monocyte activation factor and induces the expression of IL6, IL8, and TNFα while inhibiting the expression of IL10 upon activation [24]. Considering the reduced expression of 4-IBB and GITR on circulating monocytes of glioblastoma patients observed in our study, it is conceivable that monocytes shed costimulatory molecules during disease progression outlining their immunosuppressive phenotype. Despite being a key regulator of the early activation of naïve and memory T cells, CTLA-4 is expressed in B cells [25], monocytes [26,27], dendritic cells [28], and activated granulocytes [29]. Treatment with ipilimumab, a monoclonal antibody targeting CTLA-4, revealed that the clinical success of T cell immune checkpoint antibodies in patients with metastatic melanoma also relies on off-target effects via myeloid-derived suppressor cells expressing CTLA-4 [30]. The increased expression of immune checkpoint molecules on circulating myeloid and lymphoid cells in cancer patients is associated with advanced disease, and a negative prognosis independent of disease stage [31,32,33] and likely reflects the immunosuppressive network in patients suffering from glioblastoma in the present study. Besides immune modulations accompanying disease progression, the patient’s medication also alters the immune status. Dexamethason is frequently used to reduce clinically relevant brain edema typically surrounding glioblastoma tissues but has multiple adverse effects and strongly influences immune cell counts and the cytotoxic activity of T cells [34], which is in line with the suppressive immune status observed in dexamethasone-medicated patients in our study. This is particularly important as steroid-induced leukopenia likely reduces the efficacy of immunotherapies in the *per se* compromised immune compartment in glioblastoma tissues of patients. Interestingly, a marked increase in GITR expression was observed in circulating CD4^+^ T cells in glioblastoma patients in the present study. While on the one hand, GITR expression on naïve T cells is suggested to be induced following antigen-receptor stimulation, it is also highly expressed in CD4^+^ regulatory T cells. Recent research indicated that GITR signaling promotes expansion of T_reg_ cells and enhances their regulatory activity [35]. In this view, it is conceivable that the increased expression of GITR on CD4^+^ T cells is linked to increased frequencies of regulatory T cell subsets and underlines the immune dysregulation in glioblastoma patients. 

Glioblastoma is characterized by a highly immunosuppressive tumor microenvironment with a predominance of immunosuppressive myeloid cells, the release of the oncometabolite 2-hydroxyglutarate (2-HG) in IDH-wildtype glioblastoma [36], lymphopenia due to steroid treatment and chemoradiation as stated above, and multiple modes of T cell dysfunction. In newly diagnosed tumors, microglia-derived myeloid cells are predominant but outnumbered by monocyte-derived myeloid cells following recurrence, especially in hypoxic tumor environments [37]. Glioma-associated microglia/macrophages have been implicated in brain tumor angiogenesis and resistance to anti-angiogenic therapies [12] and may contribute to the colonization and outgrowth of brain metastasis [38], potentially through their ability to modulate blood vessel integrity and function [39,40]. The myeloid compartment is large, diverse, and dynamic across disease stages, limiting characterization along the linear M1/M2 phenotype axis. Notwithstanding, the anti-inflammatory/pro-tumorigenic activation of microglia/ macrophages is characterized by reduced levels of iNOS expression and NO release, impaired phagocytic abilities, elevated levels of ARG1, CD163, CD206, and several cytokines, including IL-10 and TGF-β [37]. Compared to healthy control participants, histopathological confirmation revealed an increase in parenchymal CD163^+^ microglia/ macrophages paralleled by increased expression of PSGL-1. Despite being considered a T cell immune checkpoint [41,42], PSGL-1 has been shown to aid in the spreading and metastasis of melanoma and colon cancer cells via P-selectin (SELP) mediated platelet activation [43,44]. In glioblastoma, cancer cells overexpress and oversecrete SELP to exploit PSGL-1 signaling in glioma-associated microglia/macrophages. shRNA knockdown of SELP revealed an increase in pro-inflammatory and T cell recruitment signatures compared to negative controls. Likewise, blocking SELP function was accompanied by delayed tumor growth, prolonged survival, and improved immune infiltration in vivo [44]. Interestingly, intratumoral myeloid cells were also found to express low levels of PD1. Using a conditional allele that allowed myeloid-specific (PD1^f/fLysMcre^) or T cell-specific (PD1^f/fCD4cre^) targeting of the *Pdcd1* gene, Strauss and colleagues could recently show that granulocyte/macrophage progenitors (GMPs), which accumulate during cancer-driven emergency myelopoiesis and give rise to MDSCs, express PD1 which is of high therapeutic relevance. In tumor-bearing PD1^f/fLysMcre^ mice, accumulation of GMP and MDSCs was prevented while the systemic output of effector myeloid cells was increased. In addition, myeloid cell-specific PD1 ablation increased T_em_ cells and improved their functionality, ultimately mediating anti-tumor immunity despite a perceived PD1 expression on T cells [45].

In line with previous results, CD4^+^ T_em_ was the predominant CD4^+^ T cell subset inside glioblastoma [22]. T_em_ cell subsets showed a strong upregulation of PD1, involved in functional T cell exhaustion and providing a rationale for anti-PD1 treatment of glioblastomas to restore T cell function [22]. Exhaustion describes a hyporesponsive T cell state due to chronic antigen exposure, characterized by upregulation of various co-inhibitory receptors. Previous studies indicated that glioma-derived MDSCs could induce antigen-specific CD4^+^ tolerance or T cell exhaustion in a mouse model [46], contributing substantially to dysfunction among activated T cells that successfully arrive at the tumor side. In line with our study, intratumoral T cells in glioblastoma were recently found to express multiple immune checkpoints, including PD1, TIM3, LAG3, TIGIT, and CD39, signs of a severe exhaustion signature amidst T cells [16]. Those findings support the rationale of combinatorial checkpoint blockade in this disease, already approved as first-line therapy for patients with metastatic or inoperable melanoma by the FDA since 2016 and investigated as a therapeutic option in glioblastoma [47]. Disease stage-dependent assessment of immune biomarker profiles might further serve to design individualized therapeutic strategies in neurooncology.

## 5. Conclusions

Our findings shed light on glioblastoma patients’ peripheral and intratumoral immune status, delineating the highly immunosuppressive environment compared to healthy control participants. The exhaustive signature found on T cells provides a rationale for future investigation of combinatorial ICB in glioblastoma patients. In addition, the immunosuppressive phenotype of myeloid cells indicates a promising target for successful immunotherapeutic approaches. These findings have to be elucidated in functional experiments in the future.

## Figures and Tables

**Table 1:** **Overview of patient and proband cohorts**. Summary of GBM patient and proband cohorts used for flow cytometric immunophenotyping in this study. N.A.: not applicable. IDH: isocitrate dehydrogenase. MGMT: O^6^-methylguanine-DNA-methyltransferase.

	GBM Patients	Proband Cohort
Number of patients	28	28
Mean age/range	68 (41–87)	67 (36–85)
Gender (male/female)	18/10	18/10
IDH-status (wildtype/mutant/N.A.)	29/0/6	-
MGMT status (methylated/unmethylated)	18/17	-

## Data Availability

The underlying data of the work can be retrieved from the corresponding author upon reasonable request.

## Appendix A

**Table A1:** **Previous medical history and medication.** Summary of the previous medical history and medication of the GBM patients and age- and gender-matched control probands. COPD: chronic obstructive pulmonary disease. NSAIDs: non-steroidal anti-inflammatory drugs.

Co-Morbidities	GBM Patients	Controls
high blood pressure	17	19
atrial fibrillation	3	3
hypothyreosis	3	4
diabetes mellitus	8	4
hyperuricemia	1	1
retinal detachment	0	1
glaucoma	0	3
cataract	0	1
cardiovascualr diseases	3	6
dyslipidemia	4	2
COPD	2	4
kidney insufficiency	2	0
depression	1	1
		
**medication**		
hemostasis	11	13
NSAID’s	2	2
dexamethasone	22	1
anti-epileptics	6	2
proton-pump-inhibition	15	9
statins	5	10
antihypertensives	17	19
antidiabetics	8	6
uricostatics	3	1
antibiotics	1	0
L-thyroxin	3	5
diuretics	6	9
antidepressives	3	7

**Table A2:** List of antibodies used in this study.

Target	Host	Clone	Conjugate	Cells Stained
**flow cytometry**				
CD1c	mouse	L161	APC/Cy7	myeloid/plasmacytoid DCs
CD3	mouse	HIT3a/UCHT1	PCP5.5	T cells
CD4	mouse	RPA-T4	PE-Dazzle	T_H_ cells
CD8a	mouse	HIT8a	PC7	CTLs
CD11b	rat	M1/70.15.11.5	PE	monocytes, macrophages, DCs, neutrophils
CD14	mouse	TÜK4	APC-Vio770	monocytes, macrophages
CD14	mouse	M5E2	APC/Fire750	monocytes, macrophages
CD15	mouse	W6D3	APC/Fire750	neutrophils, eosinophils
CD16	mouse	B73.1	APC/Fire750	NK cells
CD25	mouse	BC96	BV421	activated T cells
CD45	mouse	HI30	AF700	leucocytes
CD45	mouse	5B1	PerCP-Viio770	leucocytes
CD45-RO	mouse	UCHL1	FITC	activated T cells, T_cm_, T_em_
CD55	mouse	JS11	PE-Vio770	blood cells
CD56	mouse	HCD56	APC/Cy7	NK cells
CD62L	mouse	DREG-56	BV510	T_em_, T_cm_
CD69	mouse	FN50	PE	activated T cells
CD97	mouse	VIM3b	PE-Vio770	leucocytes
CD137	mouse	4B4-1	AF647	(TNFRSF9); leucocytes
CD152	mouse	BNI3	PE	(CTLA4); T cells
CD162	mouse	KPL-1	PE	(PSGL-1); leucocytes
CD162	human	REA319	FITC	(PSGL-1); leucocytes
CD163	mouse	GHI/61.1	APC	monocytes, macrophages
CD169	mouse	7-239	PE-Vio770	macrophages
CD204	human	REA460	VioBright-FITC	macrophages
CD273	mouse	MIH18	APC	(PDL2); dendritic cells, macrophages
CD276	mouse	FM276	FITC	(B7-H3); solid tumors
CD278	hamster	C398.4A	BV421	(ICOS); T cells
CD279	mouse	EH12.2H7	AF647	(PD1); T cells
CD357	mouse	108-17	AF647	(GITR); B cells, T cells
CD366	mouse	F38-2E2	BV421	(TIM-3); T_H_1
HLA-ABC	human	REA230	APC	all
HLA-DR	mouse	AC122	APC	monocytes, macrophages, DCs
**tissue staining**				
CD68	mouse	Ab955	unconjugated	macrophages
CD162	mouse	MAB9961	unconjugated	(PSGL-1); leucocytes
CD163	mouse	NBP1-30147	unconjugated	macrophages
